# Association between extremely long working hours and musculoskeletal symptoms: A nationwide survey of medical residents in South Korea

**DOI:** 10.1002/1348-9585.12125

**Published:** 2020-04-30

**Authors:** Hyoju Sung, Ja Young Kim, Ji‐Hwan Kim, Laura Punnett, Hyemin Lee, Seung‐Sup Kim

**Affiliations:** ^1^ Department of Public Health Sciences Graduate School of Korea University Seoul Republic of Korea; ^2^ Gyeonggi Public Health Policy Institute Seongnam‐si Republic of Korea; ^3^ Francis College of Engineering University of Massachusetts Lowell Lowell MA USA; ^4^ Department of Social and Behavioral Sciences Harvard T.H. Chan School of Public Health Boston MA USA

**Keywords:** medical residents, musculoskeletal pain, South Korea, working hours

## Abstract

**Objectives:**

It has been reported that South Korea ranked as one of the longest‐working nations among OECD countries. This study sought to examine the association between long working hours and musculoskeletal pain among Korean medical residents.

**Methods:**

We analyzed a cross‐sectional survey of 1,077 medical residents in South Korea. Working hours per week were categorized as follows: <60, 60‐79, 80‐99, and ≥100. Musculoskeletal pains (ie, upper limb, lower limb, and low back pain) over the past 3 months were categorized into three groups: no pain, pain without interfering with work, and pain interfering with work. Multinomial logistic regression was used to examine the association between long working hours and musculoskeletal pains after adjusting for covariates.

**Results:**

We found that the average working hours of medical resident was 85.6 hours per week in South Korea. Compared to the medical residents working <60 hours, those working ≥100 hours per week were more likely to have upper limb pain (PR: 1.77, 95% CI: 1.37, 2.30) interfering with work or low back pain (PR: 2.15, 95% CI: 1.51, 3.06) interfering with work, whereas no statistically significant association was observed in the analysis of lower limb pain.

**Conclusions:**

This study suggests that extremely long working hours are associated with upper limb and low back pain interfering with their work among Korean medical residents.

## 1 BACKGROUND

The South Korean worked an average of 1993 hours in 2018, ranked as the third longest working hours among member countries in the Organisation for Economic Co‐operation and Development (OECD).^1^ In particular, Korean medical interns/residents commonly work in excess of 90 hours per week, which is two times higher than the average of all OECD countries.^2^

Musculoskeletal pain is prevalent among medical doctors.^3^, ^4^ A previous cross‐sectional study of 1,386 Korean medical residents reported that the prevalence of upper limb, lower limb, and low back pain was 77.6%, 35.4%, and 71.3%, respectively.^2^ Another study of 125 medical residents found that the prevalence of low back pain was 56.8% in Turkey.^5^

Epidemiological studies have found that long working hours of medical doctors might negatively influence musculoskeletal pain. For example, a cross‐sectional study of 361 medical doctors in China reported an association between overtime work and musculoskeletal complaints.^6^ Among 10,922 Indian ophthalmologists, those working ≥50 hours per week were 1.65 times more likely to have low back pain compared with those working <50 hours per week.^7^ Musculoskeletal pain among doctors may influence job burden or work performance and result in lower capacity for and quality of patient care.^3^, ^8^

However, to our knowledge, no previous study about long working hours and musculoskeletal pain has been conducted in a group which has extremely long working hours such as Korean medical residents. This study sought to assess prevalence of musculoskeletal pain (ie, upper limb, lower limb, and low back pain) and to classify each pain into two types: pain interfering with work and pain without interfering with work. Furthermore, we examined how long working hours was associated with each type of the three musculoskeletal pains among medical residents in South Korea.

## 2 METHODS

### 2.1 Study design and population

A nationwide cross‐sectional survey, 2014 Korean Interns and Residents Survey, was conducted to assess demographic factors, working environments, and health status among medical interns/residents.^2^ In South Korea, a medical resident means a person who has completed an medical internship and who is under training to practice medicine at specialized medical department in a program recognized by the Minister of Health and Welfare. It is rarely possible to change medical specialty during medical residency.

The target population comprised 11 564 doctors‐in‐training whose e‐mail addresses or mobile phone numbers were registered with the Korean Intern Resident Association (KIRA). Data were collected through online surveys from August to September 2014. The response rate of our survey was 16.5% (n = 1912). We excluded individuals who did not agree to the use of their information for academic purposes (n = 5) or who were divorced or widowed due to small sample size (n = 4). Also, we excluded individuals with missing information including 374 medical interns because they do not have one specific medical specialty (n = 826). In total, 1077 medical residents were included in this study. Because each of the three outcome variables had differential response rate, each analysis was composed of different numbers of medical residents (1070 for upper limb pain, 949 for lower limb pain, and 1041 for low back pain) after excluding the individuals with missing information for each outcome. This research received approval from the Institutional Review Board of the Korea University Office of Research (1040548‐KU‐IRB‐14‐86‐A‐2).

### 2.2 Exposure and outcomes

Working hours per week was assessed using a question, “How many hours did you work last week?” The survey participants can choose from 0 to 168 hours. Most of previous studies defined the long working hours as “40 hours or more per week.” We used the different cutoff to define long working hours because the average working hours of medical residents was 86 hours per week in our survey.^9^ So, the responses were classified into four categories: <60, 60‐79, 80‐99, and ≥100 hours.

Experience of musculoskeletal pains was assessed using two yes/no questions with a graphic diagram to indicate affected body parts: (a) “Over the past 3 months, have you ever experienced pain or aching in the body parts (ie, neck, shoulder, forearm, wrist, low back, knee, ankle, and foot) on the diagram?” and (b) “If you have had pain or aching, did the pain interfere with work?” Using the first question, the musculoskeletal pain was categorized into three groups: “upper limb” (ie, neck, shoulder, forearm, or wrist); “lower limb” (ie, knee, ankle, or foot); and “low back.” Combining the responses of the two questions, three kinds of musculoskeletal pain were categorized as “no pain,” “pain without interfering with work,” and “pain interfering with work.”

### 2.3 Other covariates

We adjusted for sociodemographic (ie, age, sex, marital status, and annual income), work‐related variables (ie, specialty, training year, working region, and hospital type) in Model 1. Additionally, we examined how the association is changed when we additionally adjusted for health behaviors (ie, smoking and risky drinking), mental health (ie, psychological distress), and physical workload variables (ie, sitting, standing, walking, lifting/carrying, and pushing/pulling) for more conservative results in Model 2.

For sociodemographic variables, age was categorized into three groups: <30, 30‐35, and >35 years old. Marital status was classified into two groups (ie, single and married). For work‐related variables, medical specialty was classified into 25 groups according to the standard classifications used in South Korea. Working region was categorized into two areas: capital (ie, Seoul) and other areas. Hospital type was dichotomized into “university hospital or hospital with a bed capacity ≥500” versus “hospital with a bed capacity <500.”

For health behavior variables, self‐reported smoking status was divided into never smokers, former smokers, and current smokers. Risky drinking was defined based on the drinking frequency and volume. Following the guideline of the Ministry of Health and Welfare in South Korea, risky drinking means drinking more than 7 drinks for men or 5 drinks for women per occasion and more than two times per week.^10^

We used Kessler‐6 score to measure psychological distress.^11^ The answer was rated on a Likert scale from never (0) to always (4). Psychological stress was defined as a total score of 13 points or more.

Physical workload was assessed by the amount of time that individuals were exposed to each posture at workplace over the past week: sitting, standing, walking, lifting/carrying, and pushing/pulling. The participants could answer from “almost never/never” (0) to “all/almost all” (4), and were classified into three groups: low (almost never/never), moderate (less than half and around half), and high (more than half and all/almost all).

### 2.4 Statistical analysis

One‐way analysis of variance was applied to compare the average working hours by key covariates. Multinomial logistic regression was used to examine the association between long working hours and musculoskeletal pains (ie, upper limb pain, lower limb pain, and low back pain) after adjusting for covariates as categorical variables. Because the prevalence of musculoskeletal pains was greater than 10% in this study population, odds ratios from logistic regression model are likely to overestimate the prevalence ratios. So, we corrected the odds ratio to prevalence ratio using the Stata “adjrr” command.^12^ Results were presented as prevalence ratio (PR) with 95% confidence interval (CI). Two‐tailed p values less than 0.05 were considered statistically significant. All analyses were performed using STATA/SE 13.0 (StataCorp).

## 3 RESULTS

Table 1 shows the distributions of study population, prevalence of low back pain, and working hours per week by key covariates. The majority of medical residents was men (69.6%), 30‐35 years old (54.6%), single (56.4%), and working in a university hospital or hospital with a bed capacity ≥500 (92.1%). The average working hours per week was 85.6 (86.5 for male and 83.5 for female medical residents). The prevalence of low back pain that did not interfere with work was 24.0% (n = 250), and that of low back pain interfering with work was 34.6% (n = 360).

**Table 1:** Distribution of study population, low back pain, and working hours per week by key covariates among medical residents in South Korea in 2014^a^ (N = 1041)

Characteristics	Total	Prevalence of low back pain		Working hours per week	
		Without interfering with work	Interfering with work		
	n (%)	n (%)	n (%)	Mean (SD)	*P* value^b^
Sociodemographics					
Age					<.01
<30	291 (28.0)	86 (29.6)	81 (27.8)	89.7 (29.5)	
30‐35	568 (54.6)	123 (21.7)	218 (38.4)	85.0 (30.0)	
>35	182 (17.5)	41 (22.5)	61 (33.5)	80.9 (24.6)	
Gender					.121
Male	725 (69.6)	159 (21.9)	242 (33.4)	86.5 (29.3)	
Female	316 (30.4)	91 (28.8)	118 (37.3)	83.5 (28.6)	
Income (1,000 KRW)					<.01
20 000‐30 000	83 (8.0)	21 (25.3)	28 (33.7)	97.4 (34.6)	
30 000‐40 000	533 (51.2)	117 (22.0)	190 (35.7)	84.7 (28.7)	
40 000‐50 000	331 (31.8)	81 (24.5)	111 (33.5)	82.2 (27.3)	
>50 000	94 (9.0)	31 (33.0)	31 (33.0)	92.7 (29.1)	
Marital status					.094
Single	587 (56.4)	146 (24.9)	200 (34.1)	86.9 (29.5)	
Married	454 (43.6)	104 (22.9)	160 (35.2)	83.9 (28.5)	
Working environments					
Training year					<.01
First year	186 (17.9)	53 (28.5)	66 (35.5)	101.7 (29.8)	
Second year	240 (23.1)	48 (20.0)	87 (36.3)	91.4 (29.2)	
Third year	296 (28.4)	75 (25.3)	99 (33.5)	82.2 (27.9)	
Fourth year	319 (30.6)	74 (23.2)	108 (30.0)	75.1 (24.3)	
Hospital type					.051
University or ≥500 bed capacity	959 (92.1)	234 (24.4)	335 (34.9)	86.1 (29.1)	
<500 bed capacity	82 (7.9)	16 (19.5)	25 (30.5)	79.6 (28.1)	
Region					.848
Seoul (capital)	536 (51.5)	128 (23.9)	186 (34.7)	85.8 (28.1)	
Other areas	505 (48.5)	122 (24.2)	174 (34.5)	85.4 (30.1)	
Health behaviors					
Smoking					.755
Never smokers	793 (76.2)	196 (24.7)	281 (35.4)	85.3 (29.2)	
Former smokers	111 (10.7)	24 (21.6)	30 (27.0)	85.4 (27.5)	
Current smokers	137 (13.2)	30 (21.9)	49 (35.8)	87.4 (29.6)	
Risky drinking					.058
No	958 (92.0)	235 (24.5)	332 (34.7)	86.1 (29.3)	
Yes	83 (8.0)	15 (18.1)	28 (33.7)	79.8 (25.8)	
Mental health					
Psychological distress					<.01
No	680 (65.3)	156 (22.9)	190 (27.9)	78.5 (26.6)	
Yes	361 (34.7)	94 (26.0)	170 (47.1)	99.0 (28.8)	
Physical workloads at work					
Sitting					<.01
Low	18 (1.7)	4 (22.2)	8 (44.4)	101.1 (28.4)	
Moderate	445 (42.8)	98 (22.0)	177 (39.8)	94.3 (28.8)	
High	578 (55.5)	148 (25.6)	175 (30.3)	78.4 (27.3)	
Standing					<.01
Low	105 (10.1)	33 (31.4)	21 (20.0)	62.9 (20.8)	
Moderate	753 (72.3)	178 (23.6)	249 (33.1)	85.3 (27.8)	
High	183 (17.6)	39 (21.3)	90 (49.2)	99.8 (29.9)	
Walking					<.01
Low	223 (21.4)	63 (28.3)	55 (24.7)	73.0 (25.0)	
Moderate	755 (72.5)	173 (22.9)	269 (35.6)	87.8 (28.8)	
High	63 (6.1)	14 (22.2)	36 (57.1)	104.5 (29.8)	
Lifting/carrying					<.01
Low	649 (62.3)	158 (24.4)	191 (29.4)	80.7 (27.4)	
Moderate	372 (35.7)	90 (24.2)	157 (42.2)	93.4 (30.0)	
High	20 (1.9)	2 (10.0)	12 (60.0)	101.6 (28.9)	
Pushing/pulling					<.01
Low	694 (66.7)	166 (23.9)	207 (29.8)	81.1 (27.6)	
Moderate	316 (30.4)	79 (25.0)	137 (43.4)	94.1 (29.6)	
High	31 (3.0)	5 (16.1)	16 (51.6)	101.1 (31.2)	
Total	1041 (100)	250 (24.0)	360 (34.5)	85.6 (29.1)	

Abbreviations: SD, standard deviation.

^a^ Specialty not shown (see Appendix 1).

^b^ *P* value of one‐way analysis of variance test comparing average working hours per week by key covariates.

Among the survey participants, 13.2% were current smokers, 8.0% were risky drinkers, and 34.7% had psychological distress (Table 1). For the physical workload variable, 55.5% of medical residents took a sitting position for more than half of the working time. More than 70% of medical residents were standing or walking for between less than half and around half of working hours. About 60% of medical residents were almost never or never to do lifting/carrying or pushing/pulling during work.

As presented in Appendix 1, most common medical specialty was internal medicine (17.8%, n = 185), followed by anaesthesiology (7.6%, n = 79) and pediatrics (6.5%, n = 68). The medical specialty with the longest average working hours per week was neurosurgery (120.0 hours), followed by thoracic and cardiovascular surgery (118.5 hours), and general surgery (112.3 hours). The average working hours by medical specialty was presented in Appendix 1.

Table 2 shows the association between long working hours and musculoskeletal pain after adjusting for covariates of sociodemographic variables (ie, age, sex, annual income, and marital status), and work‐related variables (ie, training year, hospital type, region, and specialty). In Model 1, higher prevalence of upper limb pain interfering with work was observed for “80‐99 hours per week” (PR: 1.38; 95% CI: 1.06, 1.79) and “≥100 hours” (PR: 1.77; 95% CI: 1.37, 2.30) groups compared to the “<60 hours” group. Similarly, the prevalence of low back pain interfering with work was higher among medical residents worked 80‐99 hours per week (PR: 1.69; 95% CI: 1.19, 2.39) and ≥100 hours per week (PR: 2.15; 95% CI: 1.51, 3.06) compared to the “<60 hours” group. However, no association was observed in the analysis of upper limb and low back pain without interfering with work. No statistically significant associations were observed for lower limb pain regarding of whether or not it interferes with work.

**Table 2:** Association between working hours and musculoskeletal pains among medical residents in South Korea in 2014

Musculoskeletal pain	Working hours per week	Total	Pain without interfering with work					Pain interfering with work				
		n (%)	n (%)	Model 1^a^		Model 2^b^		n (%)	Model 1^a^		Model 2^b^	
				PR	95% CI	PR	95% CI		PR	95% CI	PR	95% CI
Upper limb	<60	199 (18.5)	67 (33.8)	1.00	Reference	1.00	Reference	68 (34.3)	1.00	Reference	1.00	Reference
	60‐79	239 (22.3)	77 (32.2)	1.03	0.77, 1.37	1.05	0.78, 1.41	87 (36.4)	1.11	0.85, 1.45	1.06	0.84, 1.36
	80‐99	269 (25.1)	94 (34.9)	1.10	0.83, 1.48	1.16	0.86, 1.56	119 (44.2)	1.38*	1.06, 1.79	1.22	0.96, 1.55
	≥100	364 (34.0)	88 (24.2)	0.77	0.55, 1.08	0.87	0.62, 1.24	205 (56.3)	1.77***	1.37, 2.30	1.45**	1.13, 1.85
Lower limb	<60	174 (18.3)	23 (13.2)	1.00	Reference	1.00	Reference	14 (8.1)	1.00	Reference	1.00	Reference
	60‐79	203 (21.4)	32 (15.8)	1.00	0.60, 1.67	1.01	0.61, 1.67	25 (12.3)	1.01	0.56, 1.84	0.99	0.58, 1.71
	80‐99	243 (25.6)	42 (17.3)	1.05	0.63, 1.74	0.99	0.59, 1.64	47 (19.3)	1.31	0.75, 2.30	1.06	0.63, 1.78
	≥100	329 (34.7)	59 (17.9)	1.14	0.67, 1.94	0.96	0.56, 1.65	80 (24.3)	1.43	0.79, 2.58	1.02	0.59, 1.76
Low back	<60	194 (18.6)	49 (25.3)	1.00	Reference	1.00	Reference	39 (20.1)	1.00	Reference	1.00	Reference
	60‐79	230 (22.1)	53 (23.0)	0.95	0.66, 1.36	0.93	0.65, 1.34	56 (24.4)	1.10	0.76, 1.60	1.08	0.76, 1.55
	80‐99	266 (25.6)	67 (25.2)	1.10	0.77, 1.56	1.07	0.75, 1.53	102 (38.4)	1.69**	1.19, 2.39	1.52**	1.08, 2.14
	≥100	351 (33.7)	81 (23.1)	0.98	0.66, 1.44	0.94	0.63, 1.40	163 (46.4)	2.15***	1.51, 3.06	1.82***	1.28, 2.59

Abbreviations: 95% CI: 95% confidence interval; PR, prevalence ratio.

^a^ Adjusted for age, sex, marital status, annual income, specialty, training year, working region, and hospital type.

^b^ Adjusted for the covariates in Model 1 and smoking, risky drinking, psychological distress, and physical workload (ie, sitting, standing, walking, lifting/carrying, and pushing/pulling).

* *P* < .05;

** *P* < .01;

*** *P* < .001.

Model 2 was additionally adjusted for health behavior variables (ie, smoking and risky drinking), mental health (ie, psychological distress), and physical workload (ie, sitting, standing, walking, lifting/carrying, and pushing/pulling) for more conservative estimates. Compared to the findings of Model 1, all the significant associations were attenuated but remained statistically significant except for the association between working 80‐99 hours per week and upper limb pain interfering with work in Model 2.

## 4 DISCUSSION

This study examined the association between extremely long working hours and musculoskeletal pain among South Korean medical residents. We found that working 80‐99 hours or ≥100 hours per week was statistically significantly associated with both upper limb and low back pain interfering with work compared to working <60 hours per week. These results are consistent with the findings of previous research on physicians, nurses, and general workers.^7^, ^8^, ^13^, ^14^, ^15^, ^16^

This study found that average working hours per week was 85.6 hours among medical residents in South Korea. Previous studies have reported that long working hours could be an important risk factor endangering patient safety. One study of 11,516 registered nurses found that working more than 40 hours increase the likelihood of medication error and needle stick injury.^17^ Another experimental study reported that reducing working hours of medical interns can reduce serious medical error in intensive care units.^18^ Long working hours affect time available for sleep, and inadequate sleep is a known risk factor for poor work performance.^19^ In fact, US medical residency programs have enacted strict work restrictions specifically to reduce errors due to sleep deprivation.^20^

There are several plausible pathways linking long working hours to musculoskeletal pain. First, long working hours with continued physical workload can increase fatigue. With overuse of muscles and joints, fatigue can increase the risk of musculoskeletal injury or muscle strain.^21^, ^22^, ^23^ Also, medical residents may not have enough break time to recover from occupational fatigue or exhaustion. Due to the long working hours, they do not have sufficient time to improve their physical health through activities such as physical fitness.^24^, ^25^ Additionally, chronic fatigue could increase pain sensitivity through peripheral inflammation and abnormal immune activation.^26^

In this study, the medical residents who worked 60‐79 hours per week did not have statistically significantly higher prevalence of any musculoskeletal pain compared to those working <60 hours per week. This finding might be explained by that the reference group was medical residents working less than 60 hours per week in the analysis, whereas previous long working hour studies usually used the group working less than 40 hours per week as a reference group. We chose “working <60 hours per week” as a reference group in the analysis, considering that more than 80% of our study participants worked more than 60 hours per week. The elevated prevalence of musculoskeletal pain in the reference group may explain these non statistically significant difference between the reference group and the group who worked 60‐79 hours per week in our study.

A major limitation of this study is that the cross‐sectional study design may not provide temporal information about the association between long working hours and musculoskeletal pain. However, considering South Korean cultural contexts of a hierarchical organization and system of apprenticeship training for doctors, it would not be plausible for medical resident to adjust their working hours.^27^ Further longitudinal studies are needed to examine the impact of shortened working hours on musculoskeletal pain.

Second, although this study, to our knowledge, has the largest population among medical resident health studies in South Korea, it may not be possible to apply our findings to the entire medical resident population due to the low response rate in this study. There were several reasons for this low response rate. First, the excessive working hours in this study population may be at least a partial reason for the low response rate. Furthermore, job stressors such as unreasonably high demands have been reported elsewhere as predictors of healthcare worker survey response.^28^ Given that, it is possible that a higher response rate is not feasible for South Korean medical residents. Comparison of the demographic distribution between our dataset and administrative dataset of the all medical residents is shown in Appendix 2.

Third, our findings might be vulnerable to measurement error because working hours and musculoskeletal pains were assessed through self‐reported questions. Although there are previous studies on validity of self‐reported measurement assessing working hours and musculoskeletal pain, further studies using an administrative dataset or medical records are needed to reduce measurement error.^29^, ^30^, ^31^

To our knowledge, this is the first study to assess the association between extremely long working hours, such as >100 hours per week, and musculoskeletal pain. In this study, working 80‐99 hours or ≥100 hours per week was associated with an increased prevalence of upper limb and low back pain, compared with working <60 hours per week. Further studies are needed to assess the impact of shortened working hours on positive health benefits including musculoskeletal pain.

## DISCLOSURES

*Approval of the research protocol*: This research received approval from the Institutional Review Board of the Korea University Office of Research (1040548‐KU‐IRB‐14‐86‐A‐2). *Informed consent*: All the study participants provided informed consent before starting to answer the questionnaire. *Conflict of interest*: No financial or other relationships that might lead to conflicts of interest about the publication of this material. *Registry and the registration no. of the study/trial*: N/A. *Animal studies*: N/A.

## AUTHOR CONTRIBUTIONS

HS conducted the analysis and wrote manuscript in collaboration with SSK; JYK, JHK, and SSK collected the data; JYK, JHK, LP, and HL provided the feedback and suggestions. All authors read the manuscript and approved to submission.

## Supporting information

Appendix S1

Appendix S2

## References

[1] Stat O . OECD Stat: average annual hours actually worked per worker. Available at http://stats.oecd.org/index.aspx. Accessed March 4, 2020.

[2] Kim S , Kim JY , Kim S‐S . Working condition, health and perceived patient safety among doctors in training: 2014 Korean interns & residents survey. J Korean Med Assoc. 2015;2(35):584‐607.

[3] Catanzarite T , Tan‐Kim J , Whitcomb EL , Menefee S . Ergonomics in surgery: a review. Female Pelvic Med Reconstr Surg. 2018;24(1):1‐12. 28914699 10.1097/SPV.0000000000000456

[4] Epstein S , Sparer EH , Tran BN , et al. Prevalence of work‐related musculoskeletal disorders among surgeons and interventionalists: a systematic review and meta‐analysis. JAMA Surg. 2018;153(2):e174947. 29282463 10.1001/jamasurg.2017.4947 PMC5838584

[5] Shams Vahdati S , Sarkhosh Khiavi R , Rajaei Ghafouri R , Adimi I . Evaluation of prevalence of low back pain among residents of Tabriz university of medical sciences in relation with their position in work. Turk J Emerg Med. 2014;14(3):125‐129. 27331182 10.5505/1304.7361.2014.79106 PMC4909944

[6] Smith DR , Wei N , Zhang YJ , Wang RS . Musculoskeletal complaints and psychosocial risk factors among physicians in mainland China. Int J Ind Ergon. 2006;36(6):599‐603.

[7] Venkatesh R , Kumar S . Back pain in ophthalmology: national survey of Indian ophthalmologists. Indian J Ophthalmol. 2017;65(8):678‐682. 28820152 10.4103/ijo.IJO_344_17 PMC5598177

[8] Arslan OK , Jeve Y , Doshani A . Work‐related musculoskeletal injuries amongst obstetrics and gynaecology trainees in east midland region of the UK. Arch Gynecol Obstet. 2017;296(3):489‐494. 28702699 10.1007/s00404-017-4449-y PMC5548830

[9] Bannai A , Tamakoshi A . The association between long working hours and health: a systematic review of epidemiological evidence. Scand J Work Environ Health. 2014;40(1):5‐18. 24100465 10.5271/sjweh.3388

[10] The Ministry of Health and Welfare (South Korea) . Risky drinking. Available at http://health.cdc.go.kr/health/HealthInfoArea/HealthInfo/View.do?idx=14520. Accessed March 4, 2020.

[11] Kessler RC , Andrews G , Colpe LJ , et al. Short screening scales to monitor population prevalences and trends in non‐specific psychological distress. Psychol Med. 2002;32(6):959‐976. 12214795 10.1017/s0033291702006074

[12] Norton EC , Miller MM , Kleinman LC . Computing adjusted risk ratios and risk differences in Stata. Stata J. 2013;13:492‐509.

[13] Lipscomb JA , Trinkoff AM , Geiger‐Brown J , Brady B . Work‐schedule characteristics and reported musculoskeletal disorders of registered nurses. Scand J Work Environ Health. 2002;28(6):394‐401. 12539799 10.5271/sjweh.691

[14] Trinkoff AM , Le R , Geiger‐Brown J , Lipscomb J , Lang G . Longitudinal relationship of work hours, mandatory overtime, and on‐call to musculoskeletal problems in nurses. Am J Ind Med. 2006;49(11):964‐971. 16691609 10.1002/ajim.20330

[15] Yang H , Haldeman S , Nakata A , Choi B , Delp L , Baker D . Work‐related risk factors for neck pain in the US working population. Spine. 2015;40(3):184‐192. 25384052 10.1097/BRS.0000000000000700

[16] Dembe AE , Erickson JB , Delbos RG , Banks SM . The impact of overtime and long work hours on occupational injuries and illnesses: new evidence from the United States. Int J Occup Environ Med. 2005;62(9):588‐597. 10.1136/oem.2004.016667 PMC1741083 16109814

[17] Olds DM , Clarke SP . The effect of work hours on adverse events and errors in health care. J Safety Res. 2010;41(2):153‐162. 20497801 10.1016/j.jsr.2010.02.002 PMC2910393

[18] Landrigan CP , Rothschild JM , Cronin JW , et al. Effect of reducing interns' work hours on serious medical errors in intensive care units. N Engl J Med. 2004;351(18):1838‐1848. 15509817 10.1056/NEJMoa041406

[19] Gander P , Millar M , Webster C , Merry A . Sleep loss and performance of anaesthesia trainees and specialists. Chronobiol Int. 2008;25(6):1077‐1091. 19005906 10.1080/07420520802551428

[20] Abrams RM . Sleep deprivation. Obstet Gynecol Clin North Am. 2015;42(3):493‐506. 26333639 10.1016/j.ogc.2015.05.013

[21] Almeida SA , Williams KM , Shaffer RA , Brodine SK . Epidemiological patterns of musculoskeletal injuries and physical training. Med Sci Sports Exerc. 1999;31(8):1176‐1182. 10449021 10.1097/00005768-199908000-00015

[22] Rempel DM , Harrison RJ , Barnhart S . Work‐related cumulative trauma disorders of the upper extremity. JAMA. 1992;267(6):838‐842. 1732657

[23] Kumar S . Cumulative load as a risk factor for back pain. Spine. 1990;15(12):1311‐1316. 2149209 10.1097/00007632-199012000-00014

[24] Bongers PM , de Winter CR , Kompier MA , Hildebrandt VH . Psychosocial factors at work and musculoskeletal disease. Scand J Work Environ Health. 1993;19(5):297‐312. 8296178 10.5271/sjweh.1470

[25] Sparks K , Cooper C , Fried Y , Shirom A . The effects of hours of work on health: a meta‐analytic review. JOOP. 1997;70(4):391‐408.

[26] Meeus M , Nijs J . Central sensitization: a biopsychosocial explanation for chronic widespread pain in patients with fibromyalgia and chronic fatigue syndrome. Clin rheumatol. 2007;26(4):465‐473. 17115100 10.1007/s10067-006-0433-9 PMC1820749

[27] Lim KY , Cho SM , Song HJ . The status of violence among the doctors and its relationship to authoritarianism, aggression and personality characteristics. Korean J Med Educ. 2004;16(3):299‐308.

[28] Cifuentes M , Boyer J , Gore R , et al. Job strain predicts survey response in healthcare industry workers. Am J Ind Med. 2008;51(4):281‐289. 18247321 10.1002/ajim.20561 PMC5929479

[29] Imai T , Kuwahara K , Miyamoto T , et al. Validity and reproducibility of self‐reported working hours among Japanese male employees. J Occup Health. 2016;58(4):340‐346. 27265530 10.1539/joh.15-0260-OA PMC5356941

[30] Kaergaard A , Andersen JH , Rasmussen K , Mikkelsen S . Identification of neck‐shoulder disorders in a 1 year follow‐up study. Validation of a questionnaire‐based method. Pain. 2000;86(3):305‐310. 10812260 10.1016/S0304-3959(00)00261-X

[31] Boonstra AM , Schiphorst Preuper HR , Reneman MF , Posthumus JB , Stewart RE . Reliability and validity of the visual analogue scale for disability in patients with chronic musculoskeletal pain. Int J Rehabil Res. 2008;31(2):165‐169. 18467932 10.1097/MRR.0b013e3282fc0f93

