# Supplementary material for: Association between extremely long working hours and musculoskeletal symptoms: A nationwide survey of medical residents in South Korea
Source: J Occup Health. 2020 Apr 30;62(1):e12125. doi: 10.1002/1348-9585.12125 (PMC7193152; doi:10.1002/1348-9585.12125)
Supplement: Supplementary file 1 — Appendix S1 [file JOH2-62-e12125-s001.doc]

**APPENDIX 1.**

| Appendix 1. Prevalence of lower back pain, and mean score of working hours per week by medical residents’ specialties in South Korea in 2014* | | | | | | | | |
| --- | --- | --- | --- | --- | --- | --- | --- | --- |
|  |  |  |  | Low back pain | |  | Working hours per week | |
|  |  | Total |  | Without interfering with work | Interfering with work |  |  |  |
| Specialty |  | n (%) |  | n (%) | n (%) |  | Mean (SD) | *P-value*‡ |
| Internal medicine |  | 185 (17.8) |  | 42 (22.7) | 70 (37.8) |  | 95.5 (29.4) | <0.01 |
| General surgery |  | 39 (3.8) |  | 7 (18.0) | 21 (53.9) |  | 112.3 (22.5) |  |
| Pediatrics |  | 68 (6.5) |  | 14 (20.6) | 28 (41.2) |  | 97.1 (23.6) |  |
| Obstetrics and gynecology |  | 28 (2.7) |  | 9 (32.1) | 14 (50.0) |  | 94.2 (23.8) |  |
| Neuropsychiatry |  | 56 (5.4) |  | 10 (17.9) | 11 (19.6) |  | 75.8 (23.1) |  |
| Orthopedics |  | 47 (4.5) |  | 9 (19.2) | 19 (40.4) |  | 105.2 (25.6) |  |
| Neurosurgery |  | 21 (2.0) |  | 6 (28.6) | 11 (52.4) |  | 120.0 (25.9) |  |
| Thoracic and Cardiovascular surgery |  | 8 (0.8) |  | 3 (37.5) | 3 (37.5) |  | 118.5 (26.0) |  |
| Plastic surgery |  | 21 (2.0) |  | 3 (14.3) | 5 (23.8) |  | 91.3 (28.7) |  |
| Ophthalmology |  | 37 (3.6) |  | 5 (13.5) | 9 (24.3) |  | 82.9 (28.1) |  |
| Otorhinolaryngology |  | 37 (3.6) |  | 10 (27.0) | 16 (43.2) |  | 90.9 (21.5) |  |
| Dermatology |  | 38 (3.7) |  | 7 (18.4) | 6 (15.8) |  | 58.3 (19.4) |  |
| Urology |  | 28 (2.7) |  | 4 (14.3) | 11 (39.3) |  | 95.1 (30.8) |  |
| Radiology |  | 67 (6.4) |  | 22 (32.8) | 20 (29.9) |  | 73.7 (26.9) |  |
| Radiation oncology |  | 13 (1.3) |  | 6 (46.2) | 4 (30.8) |  | 70.2 (21.3) |  |
| Anaesthesiology |  | 79 (7.6) |  | 25 (31.7) | 34 (43.0) |  | 85.2 (19.7) |  |
| Neurology |  | 27 (2.6) |  | 6 (22.2) | 12 (44.4) |  | 86.5 (29.4) |  |
| Rehabilitation medicine |  | 40 (3.8) |  | 15 (37.5) | 8 (20.0) |  | 73.8 (27.1) |  |
| Laboratory medicine |  | 16 (1.5) |  | 1 (6.3) | 5 (31.3) |  | 64.8 (13.0) |  |
| Pathology |  | 12 (1.2) |  | 4 (33.3) | 4 (33.3) |  | 65.6 (15.5) |  |
| Family medicine |  | 76 (7.3) |  | 14 (18.4) | 25 (32.9) |  | 70.9 (30.7) |  |
| Emergency medicine |  | 59 (5.7) |  | 14 (23.7) | 18 (30.5) |  | 78.3 (19.1) |  |
| Nuclear medicine |  | 10 (1.0) |  | 4 (40.0) | 2 (20.0) |  | 71.5 (19.6) |  |
| Occupational medicine |  | 29 (2.8) |  | 10 (34.5) | 4 (13.8) |  | 53.8 (16.7) |  |
| †Chi-square test for prevalence of lower back pain across specialties.  ‡One-way analysis of variance test for average working hours per week across specialties.  SD: standard deviation | | | | | | | | |
