# Supplementary material for: Association between extremely long working hours and musculoskeletal symptoms: A nationwide survey of medical residents in South Korea
Source: J Occup Health. 2020 Apr 30;62(1):e12125. doi: 10.1002/1348-9585.12125 (PMC7193152; doi:10.1002/1348-9585.12125)
Supplement: Supplementary file 2 — Appendix S2 [file JOH2-62-e12125-s002.doc]

**APPENDIX 2**

| Appendix 2. Distribution of administrative dataset and study population by demographic variables, and medical specialty. | | | | | | | | | |
| --- | --- | --- | --- | --- | --- | --- | --- | --- | --- |
| Characteristics |  | Administrative dataset (N=15,070^§^)  (N=15,865^¶^) | |  | Study population (N=1,077) | | |  | |
|  |  | n | % |  | n | | % | p-value | |
| Age |  |  |  |  |  |  | | <0.01 | |
| <30 |  | 4,130 | 27.4 |  | 298 | 27.7 | |  | |
| 30–35 |  | 7,706 | 51.1 |  | 589 | 54.7 | |  | |
| >36 |  | 3,234 | 21.5 |  | 190 | 17.6 | |  | |
| Gender |  |  |  |  |  |  | | <0.01 | |
| Male |  | 9,574 | 63.5 |  | 753 | 69.9 | |  | |
| Female |  | 5,496 | 36.5 |  | 324 | 30.1 | |  | |
| Region |  |  |  |  |  |  | | <0.01 | |
| Seoul |  | 7,274 | 48.3 |  | 644 | 52.0 | |  | |
| Other areas |  | 7,796 | 51.7 |  | 595 | 48.0 | |  | |
| Medical specialty |  |  |  |  |  | |  | | <0.01 |
| Internal medicine |  | 5,809 | 45.5 |  | 490 | | 45.5 | |  |
| Surgical Medicine |  | 4,035 | 31.6 |  | 273 | | 25.4 | |  |
| Others |  | 2,927 | 22.9 |  | 314 | | 29.2 | |  |
| ^§^Data was collected by Annual Report Membership Statistics Korean Medical Association in 2014.  ^¶^Data was collected by Korean Hospital Association in 2014. | | | | | | | | | |
